# The Effectiveness of NIRS-Based Wearable Devices in Estimating Physical Activity Intensity in Patients with Chronic Non-Communicable Diseases: A Structured Narrative Review

**DOI:** 10.3390/medsci14020317

**Published:** 2026-06-15

**Authors:** Raúl Caulier-Cisterna, Andrés Vega-Moraga, Daniel Ramos-López, Felipe Contreras-Briceño

**Affiliations:** 1 Department of Informatics and Computing, Facultad de Ingeniería, Universidad Tecnológica Metropolitana, Santiago 7800002, Chile; rcaulier@utem.cl; 2 Translational Biomedical Laboratory, Facultad de Ingeniería, Universidad Tecnológica Metropolitana, Santiago 7800002, Chile; avega@utem.cl; 3 Metabolic Rehabilitation Unit, Center of Medical Specialties, Red de Salud UC Christus, Santiago 8330033, Chile; dramosl@ucchristus.cl; 4 Instituto de Ciencias de la Salud, Universidad de O’Higgins, Rancagua 2820000, Chile

**Keywords:** wearable devices, near-infrared spectroscopy, physical activity intensity, chronic non-communicable diseases, muscle oxygenation, exercise prescription, ventilatory threshold, rehabilitation

## Abstract

**Background**: Near-infrared spectroscopy (NIRS)-based wearable devices offer non-invasive, continuous monitoring of muscle oxygenation, providing direct microvascular and metabolic information that complements indirect indices of intensity such as heart rate and accelerometry. Their clinical applicability in chronic non-communicable diseases (NCDs) remains under active development. **Methods**: A structured narrative review was conducted in PubMed, Scopus, Web of Science, and IEEE Xplore (January 2010–January 2026) using pre-specified search strings combining NIRS, muscle oxygenation, SmO_2_, StO_2_, wearable, exercise intensity, ventilatory/lactate threshold, and individual chronic disease terms. Eligible studies addressed technical validation of wearable NIRS, NIRS-derived exercise intensity estimation, clinical applications in NCDs, or rehabilitation implementation. Evidence was synthesized thematically; quality of validation studies was appraised against AMSTAR-2-informed, COSMIN-informed, or Cochrane RoB-2 criteria. **Results**: Wearable continuous-wave NIRS shows acceptable concurrent validity with frequency-domain laboratory systems (r = 0.79; range 0.69–0.88; ±8% SmO_2_ agreement in 95% of measurements) and good test–retest reliability for moderate-to-severe domains (ICC 0.72–0.91). NIRS-derived breakpoints align more reliably with the second ventilatory/lactate threshold (ICC = 0.80) than with the first (ICC = 0.53), constraining its use for prescribing lower-intensity domains. In chronic obstructive pulmonary disease, peripheral arterial disease, chronic respiratory failure and selected cardiovascular conditions, wearable NIRS detects disease-specific patterns of muscle deoxygenation and post-exercise reoxygenation that track responses to rehabilitation. **Conclusions**: Current evidence supports wearable NIRS as a complementary, intensity-aware monitoring tool—particularly for delineating the heavy/severe-intensity boundary and detecting peripheral metabolic limitations—rather than as a stand-alone replacement for ventilatory or lactate thresholds. Because much of the evidence derives from small, single-sex or athlete-only cohorts, these findings should be regarded as a promising basis requiring further validation in broader NCD populations. Implementation in NCDs requires standardized placement and calibration protocols, sex- and body composition-stratified reference values, motion-artifact mitigation, and adequately powered longitudinal trials in clinical populations.

## 1. Introduction

Chronic non-communicable diseases (NCDs)—cardiovascular disease, chronic obstructive pulmonary disease (COPD), type 2 diabetes mellitus, peripheral arterial disease (PAD), cancer survivorship and neuromuscular disorders—share, despite their pathophysiological diversity, a common functional consequence: impaired exercise tolerance secondary to either central (cardiopulmonary) or peripheral (skeletal muscle) limitations to oxygen delivery and consumption. Optimizing physical activity intensity is therefore central to rehabilitation and secondary prevention, since intensity—not volume alone—determines whether an exercise stimulus elicits the cardiovascular, metabolic and microvascular adaptations needed to reverse deconditioning. Yet, in routine clinical practice, intensity is still prescribed and monitored almost exclusively from indirect surrogates: percentage of peak heart rate, ratings of perceived exertion, METs derived from accelerometry, or cardiopulmonary exercise testing (CPET)-derived ventilatory thresholds whose translation to home-based settings is operationally demanding.

Wearable technologies that estimate physical activity intensity have proliferated, but most consumer-grade devices remain limited to accelerometry- and photoplethysmography-based inferences. These approaches estimate energy expenditure or cardiovascular strain rather than the underlying balance between local oxygen delivery and consumption, which is the variable most directly linked to exercise tolerance in patients with NCDs [1,2]. Near-infrared spectroscopy (NIRS) addresses this gap by providing a continuous, non-invasive measurement of muscle oxygen saturation (SmO_2_) and changes in oxygenated (O_2_Hb) and deoxygenated (HHb) hemoglobin/myoglobin in the microcirculation [3,4]. By directly quantifying tissue-level oxygenation dynamics, wearable NIRS can in principle distinguish moderate, heavy and severe exercise intensity domains; identify metabolic and ventilatory breakpoints; and disentangle central from peripheral limitations during ambulatory rehabilitation [5,6].

This review focuses specifically on the contribution of NIRS-based wearables to estimating physical activity intensity in patients with NCDs. We therefore distinguish, throughout, between (i) technical validation evidence; (ii) physiological evidence linking NIRS-derived variables to recognized intensity markers (gas-exchange thresholds, lactate thresholds, exercise domains); (iii) disease-specific clinical evidence; and (iv) implementation evidence in rehabilitation settings. This structured approach facilitates a more comprehensive evaluation of the translational potential of wearable NIRS technologies in chronic disease rehabilitation and exercise prescription.

### Near-Infrared Spectroscopy: Principles Relevant to Intensity Estimation

NIRS utilizes the optical properties of hemoglobin and myoglobin within the 600–1100 nm wavelength range. Differential absorption between oxygenated and deoxygenated chromophores allows estimation of local SmO_2_, tissue saturation index (TSI) and changes in O_2_Hb, HHb and total hemoglobin (tHb) [3,7]. Three instrument families dominate the field: continuous-wave (CW) devices—small, low-cost and increasingly wearable; spatially resolved spectroscopy (SRS) systems—providing absolute saturation values from arrays of detectors; and frequency-domain (FD) and time-domain (TD) systems—laboratory-grade references that quantify absorption and scattering separately and remain the gold standard for accuracy [4,8].

From an exercise physiology standpoint, the most relevant NIRS-derived quantities for intensity estimation are: (i) HHb breakpoints during incremental exercise, which co-locate with the gas-exchange threshold and the respiratory compensation point in a substantial proportion of athletes and patients [9,10]; (ii) SmO_2_ minimum and reoxygenation half-time during recovery, which reflect peripheral muscle oxidative capacity and microvascular function [11]; and (iii) the ratio between cerebral and locomotor-muscle oxygenation across intensity domains, which diverges as patients approach exhaustion and may anticipate task disengagement [12]. These mechanistic underpinnings explain why NIRS-based wearables are conceptually well suited to support intensity prescription in chronic disease populations, where conventional heart-rate-based prescription is confounded by chronotropic incompetence, beta-blockade or autonomic dysfunction.

## 2. Materials and Methods

### 2.1. Review Design

This work was conducted and reported as a structured narrative review. We did not register a protocol on PROSPERO, and we did not perform a meta-analysis; the heterogeneity of populations, devices, anatomical sites, exercise modalities and outcome metrics precluded quantitative pooling. Methodological transparency was nevertheless prioritized, in line with current expectations for narrative syntheses informing clinical recommendations.

### 2.2. Search Strategy

PubMed, Scopus, Web of Science and IEEE Xplore were searched from January 2010 to January 2026. The final search was performed on 20 January 2026. Search strings combined three blocks with Boolean operators: (a) technology—“near-infrared spectroscopy” OR “NIRS” OR “muscle oxygenation” OR “SmO_2_” OR “tissue saturation index” OR “oximetry”; (b) form factor—“wearable” OR “portable” OR “ambulatory” OR “continuous-wave”; (c) construct—“exercise intensity” OR “physical activity” OR “ventilatory threshold” OR “lactate threshold” OR “rehabilitation” OR each disease label (“COPD”, “heart failure”, “peripheral arterial disease”, “diabetes”, “chronic respiratory failure”, “cancer”, “hypertension”, “sarcopenia”). Reference lists of retrieved reviews were hand-searched to identify additional relevant studies.

### 2.3. Eligibility Criteria

Studies were eligible when they met all of the following criteria: (i) reported original data or were systematic reviews/meta-analyses; (ii) used wearable, portable or laboratory-translatable NIRS systems; (iii) addressed at least one of (a) technical validation/reliability, (b) NIRS-derived exercise intensity estimation, (c) clinical application in an NCD population, or (d) implementation/feasibility in a rehabilitation or ambulatory setting; and (iv) were peer-reviewed and available in English. Conference abstracts without full text, editorials and animal studies were excluded. A small number of foundational methodological references (pre-2010) were retained when their content remains canonical (e.g., NIRS instrumentation reviews).

### 2.4. Study Selection and Data Extraction

Titles and abstracts were screened by two reviewers independently; full-text review was performed for potentially eligible records. Discrepancies were resolved by discussion. From each included study we extracted: population (age, sex distribution, disease and severity), NIRS device type and manufacturer, anatomical placement, exercise modality, comparator (e.g., FD-NIRS, CPET-derived thresholds, blood lactate), main NIRS-derived outcome and primary finding. Disease- and intensity-specific synthesis tables (Table 1) were built from these extractions.

**Figure 1:**
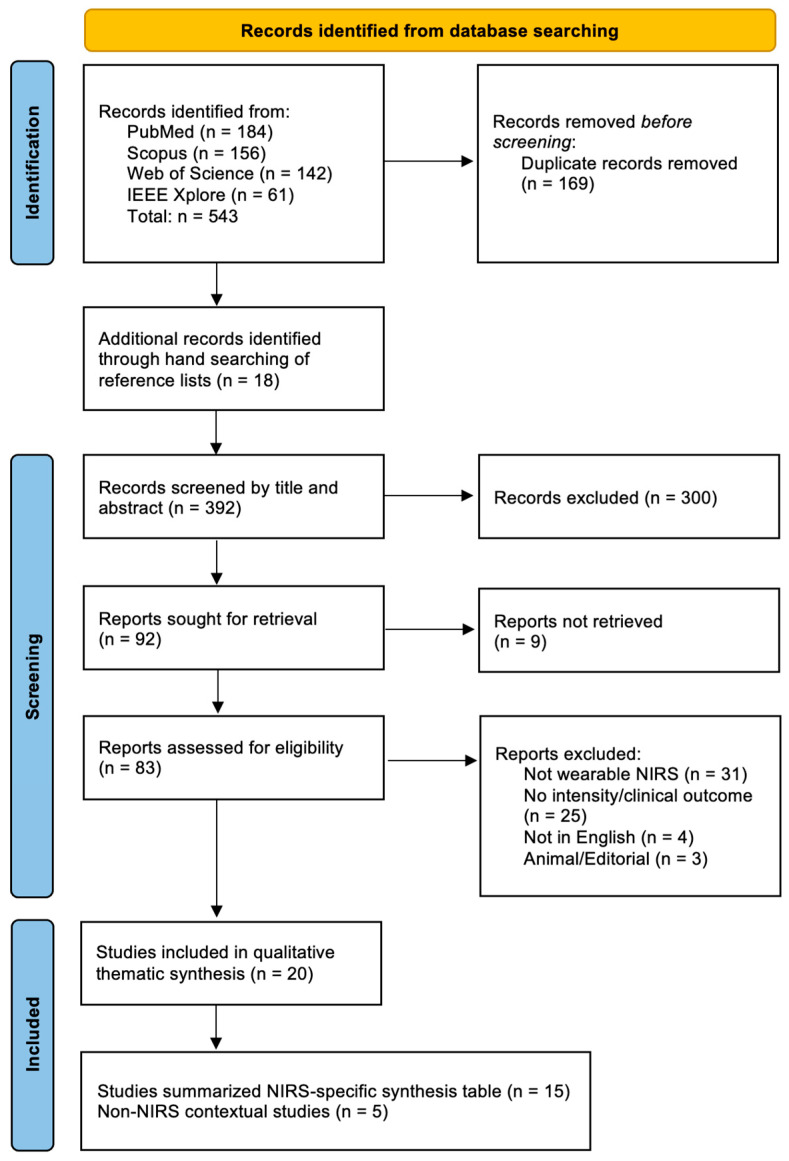
Flow diagram of the structured narrative review, summarizing the number of records identified from each database and from hand-searching, duplicates removed, records screened by title and abstract, full-text articles assessed for eligibility with reasons for exclusion, and studies finally included in the qualitative synthesis and carried forward to the evidence tables (Table 1).

The flow of records through identification, duplicate removal, title and abstract screening, full-text assessment, and final inclusion is summarized in Figure 1. Of 543 records identified through database searching and 18 through hand-searching of reference lists, 169 duplicates were removed, 392 records were screened, and 83 full texts were assessed for eligibility. Twenty studies met all eligibility criteria and were included in the qualitative synthesis: 15 NIRS-specific studies are summarized in Table 1 and were carried forward to a formal methodological-quality appraisal (Table S1, Supplementary Materials), and 5 non-NIRS studies are reported as context in Section 3.1 and Section 3.5.

### 2.5. Quality Appraisal

Given the heterogeneity of designs, a single risk-of-bias instrument was inappropriate. Validation and reliability studies were appraised against criteria informed by COSMIN [13] (clarity of construct, blinding, sample size, statistical handling, agreement metrics). Systematic reviews and meta-analyses were appraised using AMSTAR-2-informed criteria, focusing on search comprehensiveness, duplicate screening, risk-of-bias assessment of primary studies, and appropriateness of synthesis methods. Intervention studies were appraised qualitatively for risk of selection, performance and detection bias following. Intervention studies were appraised qualitatively for risk of selection, performance and detection bias following Cochrane RoB-2 principles. Rather than reporting quantitative scores, we incorporated the resulting judgements as caveats in the synthesis and explicitly flagged studies with small samples, single-sex designs, or athlete-only cohorts whose findings cannot be uncritically transferred to NCD populations. To make these judgements transparent and verifiable, the appraisal outcomes for the 15 studies carried forward are tabulated in the Supplementary Materials (Table S1), with columns indicating sample-size adequacy, the principal risk-of-bias concern, sex representation, the main external-validity limitation, and the study population (healthy participants, athletes, or clinical cohorts). This presentation allows readers to weigh the strength of the evidence underlying each thematic block directly.

### 2.6. Scope of the Search

Wearable technologies that do not use NIRS were intentionally excluded from the disease-by-disease synthesis. They are addressed only in a short contextual subsection (Section 3.5) to illustrate the broader wearable-health ecosystem in which NIRS is embedded.

## 3. Results

### 3.1. Technical Validation of Wearable NIRS Systems

Technical validation of wearable NIRS is considered here as a single, integrated body of evidence spanning four interrelated properties—concurrent validity against laboratory-grade devices, test–retest reliability, the availability of stratified reference values, and stability under motion—because these properties jointly determine the confidence that can be placed in field measurements used for intensity estimation.

With respect to concurrent validity, the concurrent validity of wearable CW-NIRS against frequency-domain systems has been examined in athletic and healthy adult cohorts. Peikon et al. [14] compared a wearable CW-NIRS sensor with a benchtop FD-NIRS device during incremental cycling in endurance-trained adults; the pooled Pearson correlation for SmO_2_ was r = 0.792 (range 0.69–0.88), and 95% of paired measurements fell within ±8% SmO_2_. An earlier wearable-validation study by Farzam et al. [9] reported correlations between CW and FD-NIRS exceeding 0.70 for both SmO_2_ and hemoglobin/myoglobin concentrations across 17 cyclists. Critically, neither device accurately predicted the lactate threshold power from raw SmO_2_ alone; only when the manufacturer’s proprietary algorithm was applied did predictions fall within half of a power increment. From a clinical perspective, an agreement of ±8% SmO_2_ is acceptable for monitoring intensity zones (where typical exercise-related SmO_2_ excursions exceed 20%) but is too coarse to detect the small, slow changes in baseline SmO_2_ that might index rehabilitation-induced microvascular adaptation.

Wearable FD-NIRS instruments have recently approached the size of CW devices. Lahade et al. [4] described a 37 g, sub-7 × 3 × 3 cm FD-NIRS module with accuracies of 0.0007 mm^−1^ for absorption and 0.08 mm^−1^ for reduced scattering, demonstrating that miniaturization need not compromise quantitative recovery of optical properties. The broadband NIRS instrumentation review by Talati and Tachtsidis [8] confirms a clear trajectory toward micro form-factor spectrometers and fiberoptic innovations that should narrow the laboratory-field gap over the next decade.

Turning to test–retest reliability across intensity domains, two recent reliability studies establish the boundaries of confidence for wearable NIRS measurements. Keller et al. [15] reported intraclass correlation coefficients (ICC) of 0.72–0.91 for SmO_2_ across the moderate-to-severe intensity range during incremental cycling in 13 physically active males but flagged limited sensitivity to small intra-individual changes. Yogev et al. [16] examined trained cyclists during severe-intensity constant-load bouts and reported an ICC of 0.87 for mean work SmO_2_, with a standard error of measurement of 12%. Taken together, these data support the use of wearable NIRS for longitudinal individual tracking across moderate-to-severe intensities, but caution against interpreting small changes (<5% SmO_2_) as clinically meaningful in a single session.

A third property concerns the availability of sex-, age- and body-composition-stratified reference values. Reference data remain a critical gap. Faria et al. [17] reported sex- and age-stratified normative percentiles for tissue oxygen saturation in the triceps surae of 288 healthy adults aged 30–79 years. Men exhibited significantly lower resting tissue oxygen saturation (StO_2_) and faster deoxygenation and reoxygenation rates than women (*p* < 0.05), with significant correlations to body mass index and body fat percentage. This is, to our knowledge, the only published normative dataset of comparable size; sex-stratified reference values for the vastus lateralis, gastrocnemius medialis and intercostal muscles—anatomical sites most relevant for cardiopulmonary rehabilitation—are not yet available. The implication for intensity estimation is non-trivial: in absence of sex- and body composition-adjusted thresholds, identical absolute SmO_2_ values may correspond to different intensity domains in different patients.

Finally, regarding motion artifact and field-based stability, Zhang et al. [18] developed an early ambulatory NIRS prototype (NINscan; Martinos Center for Biomedical Imaging, Charlestown, MA, USA) integrating NIRS, ECG, respiration and acceleration channels with motion-resistance algorithms, providing proof of concept that motion artifact can be substantially reduced by signal-processing strategies. In a field-based case report, Stöggl and Born [19] documented minimal triceps brachii SmO_2_ drift (0.027% per minute) in an elite cross-country skier during a long-distance race, confirming that wearable CW-NIRS can remain stable during prolonged outdoor exercise. The translation of these findings to ambulatory patients—who walk, climb stairs and perform activities of daily living rather than cycle on a stationary ergometer—remains underexplored.

### 3.2. Exercise Physiology and Muscle Oxygenation Monitoring for Intensity Estimation

#### 3.2.1. NIRS-Derived Breakpoints and Metabolic/Ventilatory Thresholds

The capacity of wearable NIRS to estimate physical activity intensity rests on the empirical co-location of NIRS-derived breakpoints with classical gas-exchange and lactate thresholds. Sendra-Pérez et al. [20] synthesized this evidence in a PRISMA-compliant systematic review and meta-analysis of 15 studies that compared portable NIRS thresholds with ventilatory (VT) and lactate (LT) thresholds during incremental cycling and running. Pooled ICC between the exercise intensity at the second muscle oxygenation threshold (MOT2) and the second ventilatory/lactate threshold reached 0.80, supporting wearable NIRS as a reasonable surrogate for the heavy/severe intensity boundary—arguably the most clinically relevant transition in rehabilitation prescription. By contrast, the ICC for the first muscle oxygenation threshold (MOT1) versus VT1/LT1 was 0.53 (based on only three studies), with fair-to-moderate reliability in running (ICC = 0.23–0.49) [20]. This is a substantive limitation: the moderate/heavy boundary is precisely the intensity around which many rehabilitation prescriptions are anchored. Clinically, wearable NIRS therefore appears better suited to delineate the upper rather than the lower intensity transition.

The updated systematic review by Perrey, Quaresima and Ferrari [21] reinforces this hierarchy: muscle oximetry has become one of the primary monitoring tools alongside heart rate and mechanical power, but its use as a stand-alone intensity predictor remains constrained by inter-individual variability in breakpoint detection algorithms, threshold definitions, and inter-muscle variability.

#### 3.2.2. Muscle Deoxygenation Dynamics Across Intensity Domains

The systematic review by Orcioli-Silva et al. [12] synthesized cerebral and muscle NIRS responses to exercise in healthy adults and demonstrated intensity-dependent patterns. Muscle deoxygenation (HHb) increases progressively with intensity, plateauing near voluntary exhaustion, while cerebral oxyhemoglobin first rises with intensity and then declines at exhaustion. This cerebral-muscle dissociation is intensity-specific and has been proposed as a complementary marker to detect impending task disengagement, particularly relevant in patients with cognitive frailty or limited cardiopulmonary reserve. Tuesta et al. [22] further demonstrated, in a systematic review of clinical exercise trials, that NIRS-derived microvascular adaptations track with training intensity and modality across cardiovascular, metabolic and musculoskeletal disease populations.

#### 3.2.3. Central Versus Peripheral Limitations During Incremental Exercise

**Figure 2:**
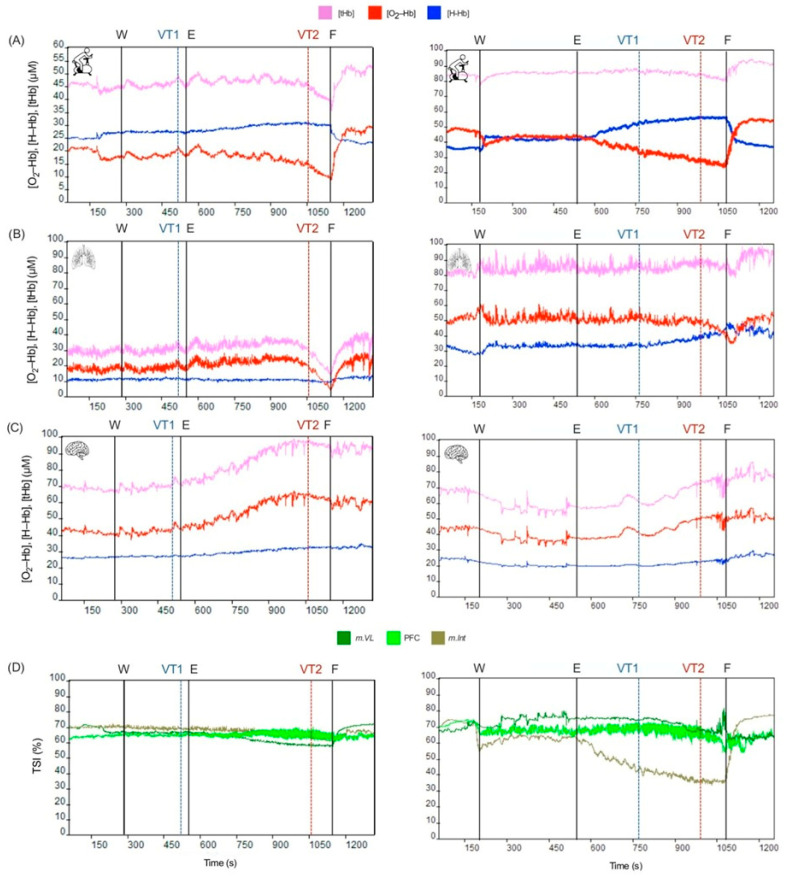
Representative NIRS-derived hemodynamic responses during incremental exercise showing central (left) vs. peripheral (right) limitations. (**A**) m.Vastus Lateralis, (**B**) m.Intercostales, (**C**) Prefrontal cortex (PFC), and (**D**) Tissue saturation index (TSI, %) throughout cardiopulmonary exercise testing. Events: W = Warm up; E = Exercise; VT1 = Ventilatory threshold 1 or aerobic ventilatory threshold; VT2 = Ventilatory threshold 2 or anaerobic ventilatory threshold; F = Finalized exercise or VO_2–max_. Adapted with permission from Ref. [23]. Copyright 2024, Carreño-Román et al.

Carreño-Román et al. [23] proposed a wearable NIRS-based protocol to identify central (cardiorespiratory) from peripheral (skeletal muscle) limitations during incremental exercise, based on the simultaneous trajectory of TSI, O_2_Hb and HHb across three anatomical sites: the vastus lateralis, the intercostal muscles and the prefrontal cortex (Figure 2).

These three tissues respond differently depending on where the primary limitation to exercise resides. In individuals with a predominantly central limitation, intercostal and prefrontal oxygenation patterns are disrupted earlier, reflecting impaired oxygen delivery to the brain and respiratory muscles before locomotor deoxygenation becomes the dominant signal. Conversely, when peripheral mechanisms are limiting, the vastus lateralis shows progressive deoxygenation with increasing workload while cerebral and respiratory oxygenation are comparatively preserved.

This approach is particularly relevant in NCDs with mixed pathophysiology—e.g., COPD with peripheral myopathy, or heart failure with reduced ejection fraction and concomitant chronotropic incompetence—where conventional indices fail to attribute exercise limitation to its underlying mechanism. The protocol has not yet been tested at scale in heterogeneous patient cohorts; its clinical yield therefore remains promising but unproven.

#### 3.2.4. NIRS Versus Heart-Rate-Based Intensity Prescription: Critical Synthesis

Whether NIRS-derived intensity prescription is clinically superior to heart-rate-based prescription in chronic disease populations remains an open question. The available evidence supports three observations. First, NIRS measures a different physiological construct (local muscle oxygen extraction) and is therefore complementary, rather than equivalent, to heart rate. Second, in conditions with autonomic dysfunction or pharmacological heart-rate modulation, NIRS may provide complementary advantages over heart rate for anchoring intensity and represents a promising alternative requiring further validation; however, no randomized trial has yet directly compared the two methods for prescribing rehabilitation intensity in COPD, heart failure or post-cardiac surgery cohorts, and any superiority therefore remains a future translational possibility rather than a demonstrated finding. Third, the ±8% SmO_2_ agreement between wearable and laboratory-grade NIRS [14], combined with a 12% standard error of measurement at severe intensities [16], implies that NIRS-based prescription should currently be conceived as zone-based rather than fine-grained, in line with the heavy/severe domain identification supported by MOT2 ICC values of 0.80 [20].

### 3.3. Clinical Applications of NIRS in Chronic Diseases

#### 3.3.1. Chronic Obstructive Pulmonary Disease and Pulmonary Rehabilitation

Szucs et al. [24] applied wearable NIRS to 40 patients with COPD before and after a 4-week inpatient pulmonary rehabilitation program. Minimum SmO_2_ during cycling increased from 42.6 ± 12.6% to 54.8 ± 14.3% (*p* < 0.01), while peak deoxygenation (SmO_2_ max) decreased from 98.0 ± 20.5% to 90.1 ± 14.3% (*p* < 0.01). These changes are physiologically consistent with improved microvascular function and oxygen consumption; however, the absence of a non-rehabilitated control limits causal inference, and the 4-week interval is short relative to the time-course of mitochondrial adaptations in COPD. Similarly, Nyberg et al. [25] evaluated low-load/high-repetition resistance exercise in 20 patients with COPD and 15 matched controls. Cardiorespiratory demand per kilogram of work was 27–122% higher in COPD; muscle deoxygenation was disproportionately increased in COPD relative to the work performed, supporting the existence of peripheral oxygen consumption impairment that conventional heart-rate-based prescription would mask.

#### 3.3.2. Respiratory–Locomotor Muscle Interactions and Adjunct Therapies

Contreras-Briceño et al. [26] employed wearable NIRS to investigate the effects of high-flow nasal cannula (HFNC) on respiratory and locomotor muscle oxygenation during high-intensity cycling in 18 non-physically active adults. HFNC attenuated intercostal-muscle deoxygenation from 5 min onward (ΔSmO_2_ in m. intercostales, *p* < 0.05) without comparable effects on vastus lateralis oxygenation. The study illustrates how wearable NIRS can probe the redistribution of oxygen delivery between respiratory and locomotor muscles—a mechanism particularly relevant to patients with COPD, heart failure or chronic respiratory failure, in whom respiratory muscle metaboreflex contributes to exercise intolerance.

#### 3.3.3. Peripheral Arterial Disease

In PAD, NIRS has been used primarily to quantify the gastrocnemius deoxygenation-reoxygenation cycle that underlies intermittent claudication. The systematic review by Tuesta et al. [22] summarized 11 trials applying NIRS to lower-extremity arterial disease, showing that supervised exercise training improved both deoxygenation kinetics during walking and reoxygenation rates during recovery. Woessner et al. [27] designed a combined dietary nitrate and exercise intervention protocol in PAD using gastrocnemius SmO_2_ as a secondary outcome to capture microvascular and metabolic adaptations not detectable from walking distance alone. Because absolute walking distance is confounded by symptom tolerance, NIRS-derived reoxygenation half-time may provide a more objective intensity-tolerance index in this population.

#### 3.3.4. Cardiovascular Conditions and Heart Failure

Suppan et al. [28] used cerebral and skeletal muscle NIRS to evaluate exercise capacity before and after transcatheter aortic valve implantation (TAVI) in severe aortic stenosis. Post-procedural improvement in exercise capacity was associated with increased cardiac index and improved oxygen consumption in both cerebral and skeletal muscle tissue, illustrating how multi-site NIRS can resolve central from peripheral contributions to functional recovery. To date, no study has integrated wearable NIRS-based intensity monitoring into multi-component cardiac rehabilitation, and the incremental value of muscle-oxygenation feedback over conventional monitoring in heart failure therefore remains to be established.

#### 3.3.5. Chronic Respiratory Failure

Sekikawa et al. [29] compared muscle oxygen consumption and blood flow in patients with chronic respiratory failure (CRF), age-matched controls and young healthy adults during handgrip exercise at 10% and 30% of maximum voluntary contraction. CRF patients exhibited significantly lower muscle oxygen consumption and paradoxically higher muscle blood flow than controls at the same relative intensity, consistent with impaired peripheral oxygen extraction at low-intensity workloads. The clinical implication is that even at intensities perceived as light, patients with CRF operate at a microvascular oxygen-extraction reserve that is substantially compromised—an effect invisible to heart-rate or accelerometry-based monitoring.

#### 3.3.6. Metabolic Diseases and Diabetes

Jones et al. [30] used NIRS-derived changes in tissue saturation index (ΔTSI%) and the muscle oxygen recovery time constant (τ) to compare skeletal muscle oxidative capacity across ethnic groups with varying cardiometabolic risk and concluded that type 2 diabetes did not fully account for the observed differences in exercise capacity, pointing to additional ethnicity-related microvascular contributions. NIRS-based oxidative capacity therefore provides a microvascular phenotyping tool that is not captured by HbA1c or by step-count-based wearables. The clinical use of NIRS as an intensity-prescription tool specifically in diabetes remains underdeveloped and constitutes a research priority.

#### 3.3.7. Mitochondrial Dysfunction: A Transversal Hallmark Across NCDs

Mitochondrial dysfunction is not a disease entity, but a pathophysiological hallmark shared across NCDs, and a unifying contribution of wearable NIRS is its non-invasive estimation of muscle mitochondrial capacity through post-exercise SmO_2_ recovery kinetics. Willingham and McCully [11] reviewed the clinical applicability of this paradigm in spinal cord injury, multiple sclerosis, peripheral artery disease, COPD and heart failure, concluding that NIRS-derived mitochondrial capacity offers a cost-effective alternative to ^31^P-MR spectroscopy. Grassi and Quaresima [3] formalized the underlying exercise physiology in skeletal muscle oxidation function and made explicit two operational constraints: subcutaneous adipose tissue thickness above approximately 20 mm essentially abolishes the muscle signal for standard CW devices, and signal interpretation requires verification of arterial occlusion completeness. Both constraints have direct implications for intensity-monitoring fidelity in obese and sarcopenic populations.

### 3.4. Implementation Challenges in Rehabilitation Settings

#### 3.4.1. User Acceptance, Workflow Integration and Longitudinal Adherence

Van Slambrouck et al. [31] reviewed wearable wireless continuous-monitoring devices in 25 studies of hospitalized high-risk patients and reported that, while continuous vital-sign monitoring was associated with reductions in in-hospital mortality, ICU admissions and rapid-response team activations, nurses were more hesitant than patients to integrate the technology into routine care. This asymmetry—high patient acceptance, lower clinician readiness—appears generalizable to NIRS-based monitoring, although NIRS-specific implementation data remain scarce. Dash et al. [32] reached a similar conclusion in the perioperative context: continuous physiological monitoring improved engagement, but training and data-accuracy concerns acted as adoption barriers.

#### 3.4.2. Data Management, Missingness and Standardization

Mobley et al. [33] documented that approximately 30% of wearable nighttime data are missing, largely from device removal, battery failure and technical malfunction, with patterns consistent with missing-at-random mechanisms. While this dataset originated from sleep tracking rather than NIRS specifically, the implication for NIRS-based intensity monitoring is structurally identical: home-based NIRS recordings will require pre-specified handling of missing data and clinically meaningful thresholds for what constitutes an interpretable session.

#### 3.4.3. Wearable-Supported Personalized Rehabilitation: Broader Feasibility Evidence

Shen et al. [34] reported significant improvements in body composition (−4.95 kg body weight, −1.68 kg/m^2^ BMI, −1.65 percentage points body fat), fasting glucose (−1.22 mmol/L) and HbA1c (−0.92%) after a 3-month personalized exercise intervention incorporating wearable smart monitoring devices in older patients with diabetes. The intervention did not isolate NIRS as the active monitoring component, and therefore the magnitude attributable to muscle oxygenation feedback specifically cannot be inferred. Bricca et al. [35] examined a 12-week personalized exercise therapy and self-management program in 228 participants with multimorbidity, reporting a reduction of −4.7 mmHg in systolic blood pressure (95% CI: −8.8 to −0.6) versus usual care; again, NIRS was not the active ingredient. These trials are best interpreted as proof-of-feasibility for personalized, technology-enabled rehabilitation rather than as evidence of NIRS-specific clinical benefit.

#### 3.4.4. Real-World Implementation Barriers: Cost, Training, Adherence and Workflow

Beyond the signal-quality constraints discussed above, several practical barriers will determine whether wearable NIRS can be adopted in routine rehabilitation. Device cost remains a consideration: although continuous-wave sensors are substantially cheaper than laboratory-grade systems, their per-unit cost still exceeds that of accelerometers and heart-rate monitors, which is relevant for programs equipping multiple patients or home-based settings. Clinician training is a second barrier, because correct optode placement, recognition of adipose-tissue attenuation, and interpretation of SmO_2_ trajectories require competencies not yet part of standard rehabilitation curricula; the reluctance of clinicians relative to patients to adopt continuous monitoring reported by Van Slambrouck et al. [31], and the training and data-accuracy concerns identified by Dash et al. [32], are both likely to apply to NIRS. Long-term patient adherence is a third barrier, as sustained home use depends on comfort, battery life, and the perceived value of the feedback, with the digital-literacy and equitable-access considerations highlighted by Murdock et al. [36] and Wang et al. [37] being particularly salient for the older and socioeconomically diverse populations typical of NCD cohorts. Finally, integration into existing workflows—data export, interoperability with electronic records, and clinician time for review—remains largely unaddressed for NIRS specifically. Acknowledging these barriers explicitly is necessary to keep expectations of near-term clinical translation realistic.

### 3.5. Complementary Wearable Approaches

To preserve the NIRS-centered focus of this review, evidence on non-NIRS wearables is summarized concisely here as context for the NIRS-specific synthesis. Ferreira et al. [38] reported moderate validity for energy-expenditure estimates from current-generation smartwatches (Spearman correlations 0.63–0.70, mean absolute percentage error 10.10–12.55%) during intermittent moderate-intensity running. Leung et al. [39] documented higher adherence to aerobic physical activity guidelines among wearable-device users with hypertension (79.3% vs. 70.3% in non-users; prevalence ratio = 1.22). Wang et al. [40] meta-analyzed wearable monitoring in atrial fibrillation and reported reduced major adverse cardiovascular events (RR = 0.66; 95% CI 0.47–0.93), all-cause mortality (RR = 0.49; 95% CI 0.29–0.85) and ischemic stroke (RR = 0.13; 95% CI 0.07–0.26). These data illustrate the broader value of the wearable-health ecosystem; they do not, however, address the question of whether NIRS-derived intensity prescription improves outcomes beyond conventional wearable monitoring. That question remains open and constitutes the principal research priority emerging from this review.

**Table 1:** Summary of key NIRS-specific studies analyzed by disease category, with device type, exercise modality, principal NIRS-derived parameters, main findings and study-level limitations. As the “Main limitation” column makes clear, much of the evidence base derives from small, single-sex, athlete-only or otherwise restricted cohorts; the findings summarized here should therefore not be overgeneralized to broader NCD populations without further validation.

Ref.	Main Limitation	Main Finding	NIRS Parameters	Exercise Modality	Device (Mode)	Design (n)	Disease/Domain
[14]	Healthy adults only; no clinical transfer	r = 0.792 (0.69–0.88); 95% within ±8% SmO_2_	SmO_2_	Incremental cycling	CW vs. FD-NIRS	Concurrent validity (n = 10)	CW vs. FD-NIRS
[9]	Athletes only; algorithm dependency limits generalization	r > 0.70 for SmO_2_ and hemoglobin/myoglobin; proprietary algorithm required for threshold prediction	SmO_2_; [Hb + Mb]	Incremental cycling	CW-NIRS vs. FD-NIRS	Cross-sectional (n = 17 cyclists)	Concurrent validity
[15]	Male-only; physically active sample	ICC 0.72–0.91; marginal sensitivity to small changes	SmO_2_	Submaximal incremental cycling	Portable CW-NIRS (Q-LAC)	Test–retest (n = 13 males)	Reliability in active adults
[16]	Trained cyclists; severe domain only	Mean work SmO_2_ ICC = 0.87; SEM = 12%	Mean work SmO_2_, ΔSmO_2_	Severe-intensity constant-load cycling	Wearable CW-NIRS	Test–retest (n = 14)	Severe-intensity reliability
[17]	Single muscle (triceps surae); healthy adults	Lower resting StO_2_ and faster kinetics in men; correlations with BMI, body fat	Triceps surae StO_2_, deoxy/reoxy rates	Rest + occlusion test	CW-NIRS	Cross-sectional normative (n = 288, 30–79 y)	Sex and age reference
[19]	Single elite athlete; not transferable to clinical populations	Triceps SmO_2_ drift only 0.027%/min over hours	Triceps brachii SmO_2_; HR; GPS	Long-distance cross-country skiing	Wearable CW-NIRS, 4 muscles	Single-case observational	Field-based endurance
[20]	High risk of bias for confounders and participant selection	ICC 0.80 for second threshold; ICC 0.53 for first	MOT1/MOT2 vs. VT1/VT2, LT1/LT2	Incremental cycling/running	Portable CW-NIRS	Systematic review/meta-analysis (15 studies)	Threshold determination
[12]	Healthy adults only; heterogeneous protocols; no NCD transfer	Intensity-dependent deoxygenation patterns; cerebral-muscle dissociation at high intensities	HHb; O2Hb; TSI (brain and muscle)	Incremental and constant-load (various)	CW-NIRS (various)	Systematic review (63 studies)	Cerebral and muscle oxygenation during exercise
[22]	Heterogeneous protocols; few sex-stratified data	Improved deoxy/reoxy kinetics with training	Gastrocnemius SmO_2_ dynamics	Supervised exercise training	Various NIRS	Systematic review (11 RCTs)	Peripheral arterial disease
[24]	No control group; short follow-up	SmO_2_ min increase from 42.6 to 54.8% (*p* < 0.01); peak deoxygenation reduced	SmO_2_ min, max, mean; tHb	Incremental cycling, 4-wk rehab	Portable CW-NIRS	Pre-post (n = 40)	COPD/pulmonary rehab
[25]	Single-session design; no intensity-prescription endpoint	Cardiorespiratory demand 27–122% higher in COPD; disproportionate peripheral deoxygenation	Δdeoxy-[Hb/Mb] in quadriceps	Low-load high-repetition resistance	CW-NIRS	Cross-sectional (COPD n = 20; controls n = 15)	COPD vs. healthy controls
[26]	Non-clinical sample; acute design	HFNC reduced intercostal deoxygenation from 5 min (*p* < 0.05)	SmO_2_ in intercostal and vastus lateralis	High-intensity constant-load cycling ± HFNC	Wearable CW-NIRS	Crossover (n = 18)	Respiratory–locomotor interaction
[28]	No randomized comparator	Improved O_2_ consumption in brain and muscle post-TAVI	Cerebral and skeletal muscle StO_2_	Cardiopulmonary exercise testing	Cerebral + muscle NIRS	Pre-post cohort	Aortic stenosis (TAVI)
[29]	Small sample; upper-limb only	Lower muscle O_2_ consumption with higher blood flow in CRF	Tissue oxygenation; blood flow	Rhythmic handgrip 10%/30% MVC	Forearm CW-NIRS	Cross-sectional (CRF n = 12; older n = 12; young n = 13)	Chronic respiratory failure
[30]	Observational; multiple unmeasured confounders	Type 2 diabetes does not fully account for ethnic differences	ΔTSI%; reoxygenation τ	Handgrip + cycling	CW-NIRS	Cross-sectional	Diabetes/ethnicity

MOT, muscle oxygenation threshold; VT, ventilatory threshold; LT, lactate threshold; MVC, maximum voluntary contraction; HFNC, high-flow nasal cannula; CRF, chronic respiratory failure; FD, frequency-domain; CW, continuous-wave; TAVI, transcatheter aortic valve implantation.

## 4. Discussion

The evidence reviewed converges on three observations that we develop critically in this section. First, wearable NIRS systems are sufficiently mature for zone-based intensity monitoring in patients with NCDs, particularly around the heavy/severe domain boundary, but their resolution remains insufficient for fine-grained prescription. Second, NIRS contributes unique information that is genuinely complementary—not redundant—to heart rate and accelerometry, especially in disease states where conventional indices are confounded. Third, despite consistent physiological signals across NCDs, robust randomized evidence that NIRS-guided exercise prescription improves outcomes beyond conventional prescription is currently lacking. We discuss the implications of these observations below.

### 4.1. What Does the Evidence Support About Wearable NIRS for Estimating Intensity in NCDs?

The strongest evidence for NIRS-derived intensity estimation is the alignment of muscle oxygenation breakpoints with the second ventilatory/lactate threshold (ICC = 0.80; [20]). This supports the use of wearable NIRS to demarcate the heavy/severe boundary, which clinically corresponds to the upper end of moderate-intensity continuous training and the lower bound of high-intensity interval training. Conversely, the moderate threshold (MOT1 vs. VT1/LT1) is identified less reliably (ICC = 0.53; [20]), limiting the use of NIRS for prescribing the low-intensity domain where many patients with severe disease must initially train. This evidence hierarchy must be incorporated explicitly into any clinical recommendation: wearable NIRS is currently a better tool for indicating when intensity becomes too high than for confirming whether intensity is high enough.

A second, structurally important point is the gap between feasibility studies and effectiveness studies. Most NIRS evidence in NCDs originates from small, single-center, often single-sex cohorts examining within-session physiological signals (Table 1). Few studies have evaluated whether long-term NIRS-guided rehabilitation modifies clinically meaningful endpoints (peak VO_2_, 6 min walk distance, exacerbation rate, mortality, quality of life) compared to standard prescription. The COPD rehabilitation evidence [24] and the multimorbidity exercise program by Bricca et al. [35] demonstrate that personalized, wearable-supported interventions can move clinical endpoints; they do not isolate NIRS as the active component.

Taken together, these positive findings define a coherent and clinically useful role for wearable NIRS rather than a merely provisional one. The technology reliably identifies the heavy/severe-intensity boundary through the alignment of the second muscle-oxygenation threshold with the second ventilatory/lactate threshold (ICC = 0.80) [20]; it provides reproducible within-individual tracking across moderate-to-severe domains (ICC 0.72–0.91) [15,16]; and, across COPD, peripheral arterial disease, chronic respiratory failure and selected cardiovascular conditions, it detects disease-specific patterns of deoxygenation and reoxygenation that conventional heart-rate or accelerometry-based monitoring cannot resolve [22,24,25,28,29]. The lower (moderate/heavy) boundary is identified less reliably (ICC = 0.53) [20], so the practical recommendation that follows is positive but bounded: wearable NIRS is well suited to flag when intensity becomes too high and is best deployed for zone-based monitoring around the heavy/severe boundary rather than for fine-grained prescription of the low-intensity domain.

### 4.2. NIRS-Specific Methodological Challenges and Their Consequences for Clinical Interpretation

Three NIRS-specific limitations have direct consequences for the interpretation of intensity data in clinical populations. The first, and arguably the most clinically consequential, is subcutaneous adipose tissue (SAT) thickness. Grassi and Quaresima [3] established that near-infrared photons must traverse SAT before reaching muscle, and that for standard CW devices operating at 30–40 mm inter-optode distances, SAT above approximately 20 mm essentially abolishes the muscle signal—rendering SmO_2_ a composite of subcutaneous and intramuscular optical properties rather than a reliable intensity index. This threshold is non-trivial in practice as a substantial proportion of patients with type 2 diabetes, heart failure and obesity will exceed it at the anatomical sites most relevant to rehabilitation. Three correction approaches have been proposed—increased inter-optode distance, the BMI-adjusted normalization strategy of Faria et al. [17], and pre-placement SAT screening via skinfold or ultrasound—but none has been validated across devices. Until SAT correction algorithms are standardized, reporting SAT thickness alongside NIRS data should be considered a minimum transparency requirement, and patients exceeding the device-specific attenuation threshold should be assessed individually before NIRS-guided prescription is attempted.

The second limitation is anatomical placement variability: differences in inter-optode distance, probe positioning relative to muscle architecture and adhesive integrity introduce inter-session error that compounds the 12% SEM at severe intensity [16]. The third is calibration heterogeneity: SmO_2_ values are not directly comparable across devices from different manufacturers because of differences in optical pathlength assumptions and proprietary algorithms. Until cross-device calibration tools are standardized, the same nominal SmO_2_ value cannot be assumed to correspond to the same intensity domain across patients, devices or longitudinal sessions [1,3].

### 4.3. Population Diversity, Sex Differences and External Validity

A recurring weakness of the wearable-NIRS literature is the limited representation of women, older adults, ethnically diverse populations and patients with severe disease. The single normative dataset providing sex- and age-stratified percentiles for tissue oxygen saturation [17] is restricted to one anatomical site (triceps surae) in healthy adults; reference values for muscles routinely interrogated in cardiopulmonary rehabilitation (vastus lateralis, intercostal muscles) are not yet available with comparable rigor. The systematic review by Sendra-Pérez et al. [20] explicitly identified sex, performance level and adipose tissue thickness as sources of variability not yet quantified across studies. Jones et al. [30] showed that NIRS-derived oxidative capacity differs across ethnic groups beyond what type 2 diabetes alone explains, suggesting that ethnicity-specific reference values may eventually be required.

The sex-specific evidence, though predominantly from non-clinical populations, is mechanistically informative. Faria et al. [17] found that men exhibited lower resting triceps surae StO_2_, with faster deoxygenation and reoxygenation than women. These values correlated with BMI and body fat, which implicates fiber type, capillary density and regional body composition as confounders of absolute SmO_2_ beyond fitness alone. Importantly, the magnitude of this sex difference is comparable to the ±8% SmO_2_ agreement interval between wearable and laboratory-grade systems [14]. Applying male-derived thresholds to female patients could therefore introduce intensity-estimation errors of clinically meaningful magnitude. Ramos-López et al. [41] extended this picture in 74 endurance-trained adults during maximal incremental cycling and identified a sex-specific dissociation across tissue compartments. Men showed greater locomotor muscle deoxygenation (ΔTSI approximately 12% lower across all intensities; *p* < 0.001, η*p*^2^ = 0.35). Women showed greater respiratory muscle oxygen extraction and an earlier plateau in prefrontal oxygenation near exhaustion, a pattern consistent with differential susceptibility to hyperventilation-induced hypocapnia. This dissociation has a direct clinical implication in patients with COPD, heart failure or chronic respiratory failure, in whom the respiratory–locomotor oxygen-delivery trade-off is already compromised. In women, respiratory muscle deoxygenation rather than vastus lateralis deoxygenation may be the primary NIRS signal of approaching the heavy/severe intensity boundary, and single-site monitoring would fail to capture it.

The implication for intensity estimation is that absolute SmO_2_ thresholds derived in athletic male cohorts should not be transferred uncritically to female patients with NCDs. Within-individual longitudinal change remains a more defensible monitoring strategy at present, and multi-site protocols encompassing both respiratory and locomotor muscles should be considered when monitoring women in rehabilitation settings.

### 4.4. From Feasibility to Implementation: A Hierarchy of Evidence in Rehabilitation

A clearer hierarchy of evidence aids interpretation across the heterogeneous designs reviewed. Phantom and concurrent-validity studies [4,14] establish that wearable devices can measure what they claim to measure. Reliability studies [16,17] establish that the same device produces consistent values across sessions. Physiological-mechanism studies [11,25,28,29] establish that NIRS variables behave as expected in disease-relevant scenarios. Single-arm or cohort intervention studies [26,34] demonstrate feasibility of integration into rehabilitation. Randomized trials with hard clinical endpoints comparing NIRS-guided to standard prescription are essentially absent. Conclusions across this hierarchy should not be conflated: positive feasibility data do not entail positive effectiveness data, and the latter remains the principal evidence gap.

### 4.5. Distinguishing Evidence from Plausible Inference

Several claims commonly made about wearable NIRS in chronic disease management are best framed as plausible inferences rather than as established findings: (i) that NIRS-guided prescription improves adherence beyond what is achievable with heart-rate monitoring; (ii) that home-based NIRS reduces healthcare utilization; (iii) that integration with machine learning algorithms enables real-time exercise-zone identification at clinically meaningful accuracy. None of these claims is currently supported by adequately powered randomized evidence in NCD populations. Recognizing this distinction is essential to avoid over-promising clinical benefits that the current evidence base cannot sustain.

### 4.6. Research and Clinical Priorities Emerging from the Evidence Base

Based on the evidence reviewed, we identify the following priorities as emerging from the literature rather than as established standards. First, internationally harmonized wearable-NIRS validation protocols should specify (a) sensor configuration; (b) anatomical placement standards for the muscles most relevant to NCDs (vastus lateralis, gastrocnemius medialis, intercostal muscles); (c) adipose-tissue correction algorithms; (d) motion-artifact rejection criteria; and (e) minimal detectable change thresholds for SmO_2_ calibrated against clinically meaningful changes in exercise tolerance. Liu et al. [42] reviewed soft, miniaturized, modular NIRS designs that may facilitate this standardization. Second, longitudinal, adequately powered randomized trials are needed comparing NIRS-guided to conventional intensity prescription on clinically meaningful endpoints in COPD, heart failure and PAD. Third, sex-, age- and body-composition-stratified reference values must be established for at least the vastus lateralis and intercostal muscles, in both healthy and NCD cohorts. Fourth, machine-learning algorithms for real-time threshold detection should be developed and validated transparently, with publication of model architectures, training data and external-validation performance. Fifth, participatory co-design with patients, caregivers and clinicians—as proposed by Murdock et al. [36]—should accompany device development, with attention to digital literacy and equitable access to avoid widening the digital health gap [37].

## 5. Conclusions

Wearable NIRS systems can be reasonably described as a maturing—rather than mature—technology for estimating physical activity intensity in patients with chronic non-communicable diseases. The available evidence supports their use for zone-based intensity monitoring around the heavy/severe domain boundary, for non-invasive characterization of peripheral oxygen consumption in conditions where conventional heart-rate-based prescription is confounded, and for tracking microvascular and mitochondrial adaptations to rehabilitation. Their advantages over indirect activity-tracker metrics are physiological rather than technological: NIRS measures what indirect indices only infer.

At the same time, current evidence does not yet justify the use of wearable NIRS as a stand-alone replacement for ventilatory or lactate thresholds in clinical prescription. Reliability is acceptable for moderate-to-severe domains but constrained for low-intensity prescription, where the moderate threshold is identified less reliably. Population diversity—particularly female, older, ethnically diverse and severely affected cohorts—remains insufficient, and randomized trials comparing NIRS-guided to conventional prescription on hard clinical endpoints are essentially absent. The combination of methodological heterogeneity, limited normative data and small sample sizes places several frequently cited claims about clinical benefit firmly within the realm of plausible inference rather than established evidence. Because much of the supporting evidence still derives from small, single-sex or athlete-only cohorts, its conclusions should be regarded as a promising basis requiring further validation rather than as established practice for broader NCD populations.

Progress in this field will require a deliberate shift from feasibility demonstrations to standardized validation protocols, sex- and body-composition-stratified reference values, motion-resistant ambulatory instrumentation, and multicenter randomized trials embedded in real-world rehabilitation programs. With these developments, wearable NIRS has the potential to become a routine complementary tool—rather than a replacement—within personalized, intensity-aware management of chronic non-communicable diseases.

## Data Availability

No new data were created or analyzed in this study. Data sharing is not applicable to this article.

## Supplementary Materials

The following supporting information can be downloaded at: https://www.mdpi.com/article/10.3390/medsci14020317/s1, Table S1: Methodological-quality appraisal of included studies (n = 18). Validation and reliability studies: COSMIN-informed; systematic reviews: AMSTAR-2-informed; intervention studies: Cochrane RoB-2; protocol paper: Protocol appraisal (RoB-2 informed). “Overall confidence” reflects transferability to NCD populations. Yellow rows = studies added in this revision.

## Abbreviations

BMI, body mass index; CI, confidence interval; COPD, chronic obstructive pulmonary disease; COSMIN, Consensus-Based Standards for Selecting Health Measurement Instruments; CPET, cardiopulmonary exercise testing; CRF, chronic respiratory failure; CW-NIRS, continuous-wave near-infrared spectroscopy; ECG, electrocardiography; FD-NIRS, frequency-domain near-infrared spectroscopy; HbA1c, glycated hemoglobin; HFNC, high-flow nasal cannula; HHb, deoxygenated hemoglobin; ICC, intraclass correlation coefficient; LT, lactate threshold; MOT, muscle oxygenation threshold; MVC, maximum voluntary contraction; NCD, non-communicable disease; NIRS, near-infrared spectroscopy; O_2_Hb, oxygenated hemoglobin; PAD, peripheral arterial disease; PPG, photoplethysmography; PRISMA, Preferred Reporting Items for Systematic Reviews and Meta-Analyses; RoB, risk of bias; RR, risk ratio; SAT, subcutaneous adipose tissue; SmO_2_, muscle oxygen saturation; StO_2_, tissue oxygen saturation; TAVI, transcatheter aortic valve implantation; tHb, total hemoglobin; TSI, tissue saturation index; VT, ventilatory threshold.
