# Supplementary material for: The Effectiveness of NIRS-Based Wearable Devices in Estimating Physical Activity Intensity in Patients with Chronic Non-Communicable Diseases: A Structured Narrative Review
Source: Med Sci (Basel). 2026 Jun 15;14(2):317. doi: 10.3390/medsci14020317 (PMC13304124; doi:10.3390/medsci14020317)
Supplement: Supplementary file 1 [file medsci-14-00317-s001.zip › medsci-4325589-supplementary.pdf]

Supplementary Material

The Effectiveness of NIRS-Based Wearable Devices in Estimating Physical Activity Intensity in Patients with Chronic Non-Communicable Diseases: A Structured Narrative Review

Raul Caulier-Cisterna, Andres Vega-Moraga, Daniel Ramos-Lopez, Felipe Contreras-Briceno

Table S1. Methodological-quality appraisal of included studies (n = 18). Validation and reliability studies: COSMIN-informed; systematic reviews: AMSTAR-2-informed; intervention studies: Cochrane RoB-2; protocol paper: Protocol appraisal (RoB-2 informed). "Overall confidence" reflects transferability to NCD populations. Yellow rows = studies added in this revision.

Study (Ref.)	Design / sample	Population type	Sample-size adequacy	Principal risk-of-bias concern	Sex representation	External-validity limitation	Appraisal basis	Overall confidence
Peikon et al. [14]	Concurrent validity (n = 10)	Healthy / endurance-trained	Limited (small n)	Single laboratory; no blinding of analysis	Not reported / likely male-predominant	Athletes only; no clinical transfer	COSMIN-informed	Moderate
Farzam et al. [9]	Validation (n = 17 cyclists)	Athletes	Limited (small n)	Algorithm-dependent threshold prediction	Not reported	Athletes only; cycling only	COSMIN-informed	Moderate
Keller et al. [15]	Test-retest (n = 13)	Healthy / active	Limited (small n)	Marginal sensitivity to small change	Male-only	Single sex; active sample	COSMIN-informed	Moderate
Yogev et al. [16]	Test-retest (n = 14)	Athletes	Limited (small n)	Severe domain only (SEM 12%)	Not reported	Trained cyclists; one domain	COSMIN-informed	Moderate
Faria et al. [17]	Normative (n = 288, 30-79 y)	Healthy adults	Adequate	Single muscle site	Sex-stratified (both sexes)	Triceps surae only; healthy adults	COSMIN-informed	Moderate-High
Stoggl & Born [19]	Single-case field	Elite athlete (n = 1)	Inadequate (n = 1)	Anecdotal; no statistics	Single male	Single elite athlete	COSMIN-informed	Low
Sendra-Perez et al. [20]	SR / meta-analysis (15 studies)	Mixed (mostly healthy/athlete)	Adequate (pooled)	High RoB for confounders & selection	Underreported across studies	MOT1 based on few studies	Cochrane RoB-2 (qualitative)	Moderate
Orcioli-Silva et al. [12]	Systematic review (63 studies)	Healthy adults	Adequate (pooled)	Heterogeneous exercise protocols; no NCD populations included	Mixed; not stratified	Findings restricted to healthy adults; no clinical transfer	AMSTAR-2-informed	Moderate
Tuesta et al. [22]	SR / meta-analysis (11 RCTs)	Clinical (PAD)	Adequate (pooled)	Protocol heterogeneity; few sex-stratified analyses	Mixed; not stratified	PAD populations only; mixed NIRS protocols	AMSTAR-2-informed	Moderate
Szucs et al. [24]	Pre-post (n = 40)	Clinical (COPD)	Limited	No control group; short follow-up	Mixed; not stratified	No randomised comparator	Cochrane RoB-2	Low-Moderate
Nyberg et al. [25]	Cross-sectional (n = 20 + 15)	Clinical (COPD) + controls	Limited	Single-session; no prescription endpoint	Mixed; not stratified	Acute design only	Cochrane RoB-2	Low-Moderate

Study (Ref.)	Design / sample	Population type	Sample-size adequacy	Principal risk-of-bias concern	Sex representation	External-validity limitation	Appraisal basis	Overall confidence
Contreras-Briceno et al. [26]	Crossover (n = 18)	Non-clinical adults	Limited	Acute design; non-clinical	Mixed; not stratified	Healthy sample; acute	Cochrane RoB-2	Moderate
Suppan et al. [28]	Pre-post cohort	Clinical (aortic stenosis / TAVI)	Limited	No randomised comparator	Mixed; not stratified	Single-centre cohort	Cochrane RoB-2	Low-Moderate
Sekikawa et al. [29]	Cross-sectional (n = 12 + 12 + 13)	Clinical (CRF) + controls	Limited (small n)	Upper-limb only; small n	Not stratified	Handgrip only; small sample	Cochrane RoB-2	Low-Moderate
Jones et al. [30]	Cross-sectional	Clinical (type 2 diabetes) + ethnic comparators	Limited	Multiple unmeasured confounders; no NIRS standardization	Mixed; not stratified	Observational; ethnic differences not fully controlled	COSMIN-informed	Low-Moderate

Abbreviations: AMSTAR-2, A Measurement Tool to Assess Systematic Reviews (version 2); COPD, chronic obstructive pulmonary disease; COSMIN, Consensus-Based Standards for the Selection of Health Measurement Instruments; CRF, chronic respiratory failure; MOT1, first muscle-oxygenation threshold; N/A, not applicable; PAD, peripheral arterial disease; RoB-2, Cochrane Risk of Bias 2 tool; SEM, standard error of measurement; SR, systematic review; TAVI, transcatheter aortic valve implantation.
Note: Where a study did not report participant sex, this is indicated as "not reported" rather than inferred. This table should be interpreted alongside the study-level limitations in Table 1 of the main text.
